# Financial Incentive Increases CPAP Acceptance in Patients from Low Socioeconomic Background

**DOI:** 10.1371/journal.pone.0033178

**Published:** 2012-03-30

**Authors:** Ariel Tarasiuk, Gally Reznor, Sari Greenberg-Dotan, Haim Reuveni

**Affiliations:** Sleep-Wake Disorders Unit, Faculty of Health Sciences, Department of Physiology, Soroka University Medical Center, Ben-Gurion University of the Negev, Beer-Sheva, Israel; Université de Montréal, Canada

## Abstract

**Objective:**

We explored whether financial incentives have a role in patients′ decisions to accept (purchase) a continuous positive airway pressure (CPAP) device in a healthcare system that requires cost sharing.

**Design:**

Longitudinal interventional study.

**Patients:**

The group receiving financial incentive (n = 137, 50.8±10.6 years, apnea/hypopnea index (AHI) 38.7±19.9 events/hr) and the control group (n = 121, 50.9±10.3 years, AHI 39.9±22) underwent attendant titration and a two-week adaptation to CPAP. Patients in the control group had a co-payment of $330–660; the financial incentive group paid a subsidized price of $55.

**Results:**

CPAP acceptance was 43% greater (p = 0.02) in the financial incentive group. CPAP acceptance among the low socioeconomic strata (n = 113) (adjusting for age, gender, BMI, tobacco smoking) was enhanced by financial incentive (OR, 95% CI) (3.43, 1.09–10.85), age (1.1, 1.03–1.17), AHI (>30 vs. <30) (4.87, 1.56–15.2), and by family/friends who had positive experience with CPAP (4.29, 1.05–17.51). Among average/high-income patients (n = 145) CPAP acceptance was affected by AHI (>30 vs. <30) (3.16, 1.14–8.75), living with a partner (8.82, 1.03–75.8) but not by the financial incentive. At one-year follow-up CPAP adherence was similar in the financial incentive and control groups, 35% and 39%, respectively (p = 0.82). Adherence rate was sensitive to education (+yr) (1.28, 1.06–1.55) and AHI (>30 vs. <30) (5.25, 1.34–18.5).

**Conclusions:**

Minimizing cost sharing reduces a barrier for CPAP acceptance among low socioeconomic status patients. Thus, financial incentive should be applied as a policy to encourage CPAP treatment, especially among low socioeconomic strata patients.

## Introduction

Continuous positive airway pressure (CPAP) is the treatment of choice for obstructive sleep apnea (OSA) [1]–[6]. CPAP is the most effective and cost-effective treatment for OSA when compared with conservative/usual care and placebo [7]–[10]. If used on a regular basis, CPAP can effectively decrease daytime sleepiness [3]–[5], [8], reduce cardiovascular morbidity [6], [11]–[13] and health care utilization [14]. Yet many patients, mainly of lower socioeconomic (SES) background, do not accept (purchase the device) CPAP in a healthcare system that requires cost sharing [15], [16]. Factors influencing CPAP acceptance are multi-factorial and include awareness, physician involvement, support programs, spouse involvement, and health care system policies [3], [15]–[24]. Despite the increase in CPAP treatment options (i.e., bi-level positive airway pressure, auto-adjusting CPAP), treatment acceptance and adherence are low. It has been estimated that 15–30% of patients do not accept CPAP treatment from the outset [5], [24]. Patients with low SES background are less likely to commence CPAP treatment [16]. Low SES per se has been identified as a barrier to diagnosis in many previous studies outside the field of sleep [25]–[27]. When OSA patients were offered CPAP treatment in a health system that provides free access to diagnosis and titration studies, the effect of SES as the range of income is not truncated by limited access to care. Under circumstances where access to diagnosis is not limited, SES plays a role in the patient′s decision on whether to pursue a treatment in a health system that requires cost sharing [15], [16]. The CPAP device is relatively expensive; many low-income patients view this aspect of healthcare as an unnecessary expense and only 40% of those recommended for CPAP accept the treatment. For each increase in income level category, the odds for CPAP acceptance increased by 140% [16]. Patients that accept CPAP are older, have higher SES level, severe apnea, no bed partner, and heard about positive CPAP experiences from family and friends [15], [16].

Cost sharing (or co-payment) is an accepted expenditure-control strategy. It reduces the “moral hazard” phenomena (i.e., individuals with health insurance will overuse health services because they bear no portion of the financial burden) to lower consumer demand in every health care system studied [28]. On the other hand, this policy could lead to under-use of essential medications [28]–[32] and to increased use of emergency department visits and hospital days [28], [30], [32]. Such cost-related restriction could be a mechanism for worse health outcomes among low-income and other vulnerable populations who lack adequate insurance coverage [29]. In order to balance the demands for access to pharmaceuticals with pressures to constrain costs, levels of cost sharing must be set in a manner that achieves appropriate clinical and financial outcomes. Financial incentive polices in the health care system has been proposed as a strategy to promote high-value health care based on the potential for clinical benefit [33], [34] and minimize health risk-behavior [33]–[36].

Little is known about the effect of financial incentive policy on CPAP acceptance in a health care system that uses cost-sharing strategy. We hypothesized that providing a financial incentive will overcome the barrier of cost, increasing CPAP acceptance mainly among low SES OSA patients. In this study we explored the effect of financial incentive on OSA patients′ decisions to accept CPAP treatment in a healthcare system that requires mandatory cost sharing.

## Methods

### Study design

Longitudinal interventional study, time block randomization protocol (to adjust for time-dependent effects), beginning February 2009 for 24 months.

### Participants

We recruited symptomatic adult OSA patients in the working age range (ages ≤25 –<68 years, naïve to CPAP), requiring CPAP treatment [2]: apnea hypopnea index (AHI) ≥30 (events/hour) or an AHI ≥15 (events/hour) accompanied by symptoms of excessive daytime sleepiness according to an Epworth Sleepiness Scale (ESS) score ≥10 [37], and/or documented cardiovascular disease/hypertension. We excluded patients with mild OSA, requiring bi-level pressure, or language comprehension difficulties.

The Human Subject Committee of Soroka University Medical Center approved protocol number 10262. Written informed consent was obtained from all patients. CPAP acceptance was determined at the conclusion of the adaptation period, using downloaded data from CPAP devices to confirm objective CPAP use.

### Cost sharing

To initiate CPAP treatment patients were required to pay a mandatory out-of-pocket co-payment, according to the National Health Insurance Law. Cost sharing is 25–50% of CPAP cost (average cost of a CPAP device was $1320), depending on the patient′s supplementary health insurance coverage. Patients in the control group were required to pay the full cost sharing for the CPAP treatment in the range of $330 to $660, depending on their supplementary medical insurance coverage. The financial incentive group was offered CPAP at a subsidized price of $55, i.e., the ceiling for monthly out-of-pocket expenditure for drugs in patients with chronic diseases in Israel (drug expenditure above this ceiling is reimbursed according to the law). In cases where auto-titrating CPAP was purchased, patients were required to pay the difference (Methods S1).

### Socioeconomic status

Socioeconomic status classified by the researchers to one of three categories, according to self-reported monthly income: below (<20%), equal to (±20%), or above (>20%) the average monthly gross income level in Israel. The lowest group was defined as belonging to the low SES strata [15], [16], [26].

### Procedures and Questionnaires

**Figure 1:**
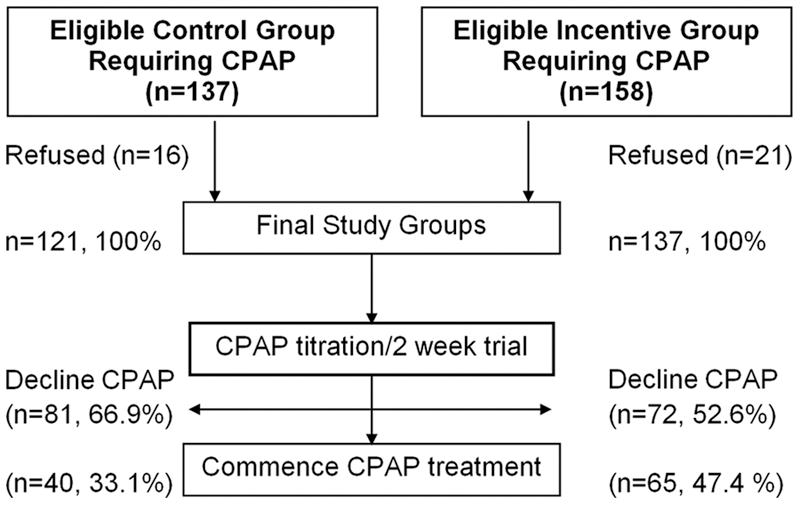
Flow chart showing stages of the diagnostic and therapeutic process and number of patients at each stage.

Initially, the control group was recruited (n = 93), followed by the financial incentive (n = 137) group (Figure 1). To adjust for time, 28 (30%) additional control subjects were recruited at the conclusion of the study. Thus, the final control group included 121 patients. All patients underwent similar protocol treatment and data handling. During the diagnostic and therapeutic process patients completed a variety of questionnaires [26], [37], [38]. A telephone survey was conducted one year following CPAP treatment initiation, to collect the following information: self-reported CPAP use (hours per night, days per week), ESS score, CPAP side effects score [39], reasons for using or declining CPAP, and social support.

### 

#### Overnight PSG

Overnight PSG was performed according to previously described methods [26]. Subjects reported to the laboratory at 8∶30 PM and were discharged the following morning; they were encouraged to maintain their usual daily routine and to avoid any caffeine and/or alcohol intake on the day of the study. Shift workers did not perform the PSG study in the week following shift duty. Upon receiving results of the CPAP titration study, sleep specialists encouraged patients to undergo a mandatory two-week adaptation period in order to commence treatment, and were informed that if they will accept (will regularly use CPAP) this treatment it will require out-of-pocket payment, according to the Israel National Health Insurance Law. In the case of the incentive group, the out-of-pocket payment was subsidized by the study. CPAP titration was performed two weeks following PSG using attendant auto-titration CPAP without concomitant PSG monitoring.

### CPAP support

A two-week period of adaptation with an auto-titrating CPAP device at home was encouraged [16], [21]. Patients were encouraged to try a variety of masks and CPAP manufacturers free of charge. At the conclusion of the CPAP support patients were offered the purchase of regular or auto-titrating CPAP (from a variety of manufacturers) as needed, they completed CPAP usage side effects [39], knowledge, and apnea beliefs scale [40], and reasons for CPAP acceptance or non-acceptance questionnaires [16].

### Data and statistical analysis

Data were analyzed using SPSS v17 Software (IBM Corporation, Somers, NY). Logistic regression analysis was used to investigate factors influencing CPAP acceptance. The null hypothesis was rejected at the 5% level (see Methods S1).

## Results

### Patients

158 and 137 patients in the financial incentive and control groups, respectively, met the inclusion criteria (Figure 1). Sixteen patients in the control group and 21 in the financial incentive group refused to be included in this study, and were excluded. The characteristics of the additional 28 control group patients recruited at the conclusion of the study were similar to the 93 subjects recruited at the beginning of the study by income level (*p* = 0.215), age (*p* = 0.404), gender (*p* = 0.667), AHI (*p* = 0.176), BMI (*p* = 0.867), and percent of subjects accepting CPAP (*p* = 1.00).

No significant differences were found between financial incentive and control groups′ characteristics, supplementary health insurance coverage, and OSA severity except for tobacco smoking (*p* = 0.003), percent of sleeping time in which oxygen saturation was below 90% (T_90%_, *p* = 0.04), which were higher in the control group (Table 1). Side effects score was similar among smoking and non-smoking patients requiring CPAP (*p* = 0.319). Smoking history did not affect the decision to accept CPAP in either group (*p* = 0.489).

**Table 1:** Comparison of control and incentive groups.

	Control group (n = 121)	Incentive group (n = 137)	*P*
CPAP acceptance (%)	33.1	47.4	0.02
Males (%)	79.3	78.8	1.0
Age (years)	50.9±10.3	50.8±10.6	0.95
BMI (kg/m^2^)	32.1±6.7	32.6±5.6	0.59
AHI (events/h)	39.9±22.0	38.7±19.9	0.62
T_90_ (%)	11.9±18.8	7.5±15.2	0.04
ESS (score)	10.1±4.9	10.2±5.3	0.89
Tobacco smoking (pack/year)	23.6±26.6	13.2±24.3	0.003
Education (years)	13.2±3.7	13.5±3.0	0.58
HTN/CVD (%)	48.8	51.8	0.71
Living with a partner (%)	86.8	85.4	0.86
Employed (%)	71.1	75.2	0.48
**Income level**			
Low (%)	40.5	46.7	0.16
Average (%)	43.0	44.5	
High (%)	16.5	8.8	

AHI – Apnea-Hypopnea Index, BMI – Body Mass Index, CPAP accepting – patients who purchased CPAP and commence treatment. CVD – cardiovascular disease, ESS – Epworth Sleepiness Scale, HTN – hypertension, T_90_ – percent sleeping time in which oxygen saturation was below 90%. Values are mean±SD.

The number of subjects ≤40 years old was similar (*p* = 0.326) in both groups, 24/137 (17.5%) and 22/121 (18.2%), respectively. Thirty-three percent (n = 40) and 47% (n = 65) in the control and financial incentive groups, respectively, accepted CPAP (*p* = 0.02; Table 1). The proportion of patients accepting CPAP among young adults was lower in both the control and incentive groups [5/22 (22%) and 6/24 (25%), respectively] compared with the entire group CPAP acceptance rate. Twenty-three percent and 29% (*p* = 0.502) in the control and incentive groups, respectively, reported that they received positive information from family and/or friends treated with CPAP, compared with 14.8% and 28.2% (*p* = 0.049) of CPAP-declining patients in the control and incentive groups, respectively. The characteristics of patients accepting and declining CPAP in the incentive and control groups are summarized in Table 2. Among patients who purchased CPAP, a greater proportion of low SES patients was found in the incentive group compared to the control group, 53.8% vs. 30%, respectively (*p*<0.01). Among those who accepted CPAP, 25% and 28% of the patients in the control and incentive groups, respectively, purchased auto-titrating CPAP and the remainder purchased the standard device. Similar CPAP pressure and post-treatment AHI were found in patients declining CPAP compared with patients accepting a regular CPAP device in both the control and financial incentive groups (Table 2). Similar side effects score, apnea knowledge, and apnea belief scale were found in both the control and incentive groups. In both groups, upon completion of the CPAP support patients declining CPAP experienced a greater side effect score (values are median and range) than patients accepting CPAP, 17 (0–63) vs. 7 (0–63), respectively (*p*<0.001). Patients declining CPAP reported less apnea knowledge compared with patients accepting CPAP 5 (0–20) vs. 6 (0–20) respectively (*p* = 0.021) and reported a lower apnea belief scale 73 (24–120) vs. 89 (24–120) respectively (*p*<0.001). Common reasons for CPAP acceptance among the control and incentive groups were: solving snoring problems (81.8% vs. 85.5% of the respondents, *p* = 0.737), reducing daytime sleepiness (72.7% vs. 78.1%, *p* = 0.575), encouragement by their partner (54.5 vs. 54.7%, *p* = 1.0), improving sleep (45.5% vs. 57.8%, *p* = 0.333), and “It is the best treatment available” (31.8% vs. 56.3%) respectively (*p* = 0.082), respectively. Common reasons for declining CPAP treatment among control and incentive groups were: “could not adapt” (100% vs. 95.2%, *p* = 1.0), “side effects” (75% vs. 66.7%, *p* = 1.0), and “CPAP cost is too expensive for me” (50% vs. 4.8%) respectively (*p* = 0.013).

**Table 2:** Characteristics of patients declining and accepting CPAP in the control and Incentive groups.

	Control group		Incentive group	
	Declined CPAP (N = 81, 66.9%)	Accepted CPAP (N = 40, 33.1%)	Declined CPAP (N = 72, 52.6%)	Accepted CPAP (N = 65, 47.4%)
Males (%)	74.1	90.0	80.6	76.9
Age (years)	51.1±11.8	50.3±8.3	47.1±10.1	54.4±9.9†
BMI (kg/m^2^)	31.2±6.5	32.5±4.8	31.2±5.3	33.5±5.4
AHI (events/hour)	37.3±20.1	45.3±23.4*	36.1±20.5	41.5±19.1*
ESS (score)	9.9±4.8	10.3±4.9	10.7±5.3	9.5±5.3
Tobacco Smoking (pack/year)	22.8±24.7	25.5±28.1	9.7±19.7	17.0±28.2‡
HTN/CVD prevalence (%)	50.6	45.0	47.2	56.9
Live with Partner (%)	84.0	92.5	84.7	86.2
Income (low, average/high)				
Low (%)	45.7	30.0	40.3	53.8+
Average/High (%)	54.3	70.0	59.7	46.2+
CPAP pressure (cmH_2_O)	8.4±2.6	9.0±2.4	8.2±2.2	8.7±2.8
AHI on CPAP (events/hour)	5.2±2.3	5.3±2.5	5.2±2.7	3.3±2.8

AHI – apnea-hypopnea index, BMI – body mass index, CPAP – continuous positive airway pressure, CVD – cardiovascular disease, ESS – Epworth Sleepiness Scale, HTN – hypertension, Income – individual monthly income relative to average monthly income level in Israel.

*p<0.05,

† *p*<0.001 comparing CPAP declined with CPAP accepting in each group.

‡ *p*<0.05 comparing CPAP accepting patients in the control and incentive groups.

+*p*<0.05 comparing CPAP accepting and CPAP declined in the incentive group.

Values are mean±SD.

Compared with average/high income patients, low-income patients included more women (*p* = 0.005), fewer patients living with a partner (*p* = 0.01), fewer years of education (*p* = 0.001), and a trend for higher BMI (*p* = 0.06) (Table 3). Odds for cardiovascular disease and hyperlipidemia were significantly higher among low-income patients (Table 3).

**Table 3:** Patient Characteristics According to Income Level.

	Low Income (N = 113)	Average/High Income (N = 145)	PV
Age (years)	51.8±10.5	50.1±10.4	0.2
Men (%)	70.8	85.5	0.005
BMI (kg/cm^2^)	33.2± 7.2	31.7 ± 5.2	0.06
AHI (events/hr)	37.4±20.8	40.7±20.9	0.22
T_90_ (%)	10.2±20.9	9.0±13.3	0.60
ESS (score)	10.7±5.4	9.7±4.8	0.11
Living with Partner (%)	79.6	91.0	0.01
Tobacco smoking (pack/years)	16.3±24.5	17.9±26.3	0.64
Education (years)	11.9±3.2	14.4±3.1	<0.001*
Education≥12 years (%)	60.6	92.4	<0.001*
**Co-morbid diagnoses**	**Prevalence**	**Prevalence**	**OR (95% CI)**
CVD (%)	57.5	44.8	1.67 (1.01–2.74)
HTN (%)	55.1	43.1	1.62 (0.78–3.37)
Hyperlipidemia (%)	57.1	37.5	2.22 (1.06–4.66)
Diabetes (%)	18.4	9.7	2.09 (0.72–6.05)

AHI – Apnea-Hypopnea Index, BMI – Body Mass Index, CVD – Cardiovascular Diseases, ESS – Epworth Sleepiness Scale, FOSQ – Functional Outcomes of Sleep Questionnaire, HTN – Hypertension, T_90_ – percent sleeping time in which oxygen saturation was below 90%. Values are mean±SD.

### Effects of financial incentive on CPAP acceptance

Multivariable logistic regression (adjusting for income level, gender, BMI, T_90%_, ESS, tobacco smoking) revealed that CPAP acceptance was determined by (OR, CI% 95) age (yr +1) (1.033, 1.004–1.064), AHI (30 vs. <30) (2.6, 1.325–4.967), and low income*financial incentive (2.9, 1.03–8.19); area under the ROC curve was 71.2%.

To explore the impact of financial incentive on CPAP acceptance, a multivariable regression analysis was performed separately for low-income patients and for average/high-income patients (Table 4). For low-income patients (adjusting for age, gender, BMI, living with partner, tobacco smoking) CPAP acceptance was sensitive to financial incentive (y/n) (3.43, 1.09–10.85), age (1.1, 1.03–1.17), AHI (30 vs. <30) (4.87, 1.56–15.2), and family/friends having positive experience with CPAP (y/n) (4.27, 1.05–17.51); area under the ROC curve of 81%. Adjusting for side effects score, apnea belief scale, and apnea knowledge score did not affect our findings that financial incentive is an independent predictor of CPAP acceptance among low-income patients. Among average/high-income patients CPAP acceptance was sensitive to AHI (30 vs. <30) (3.16, 1.14–8.75) and living with partner (y/n) (8.82, 1.03–74.8); area under the ROC curve 69.3%. T_90%_ was associated with AHI and smoking (*p*<0.001); education and smoking were associated with income level (*p*<0.001), and cardiovascular disease/hypertension prevalence was associated with age (*p* = 0.003). Therefore, they were not included in the model.

**Table 4:** Determinants of OSAS Patients Accepting CPAP Treatment among Low Income and Average/High Income Strata.

Variable	Low Income (N = 113)			Average/High Income (N = 145)		
	OR	95% CI	Pv	OR	95% CI	Pv
Financial Incentive (yes vs. no)	3.43	1.09–10.85	0.036	1.07	0.44–2.61	0.882
Age (+1 year)	1.10	1.03–1.17	0.002	1.02	0.98–1.06	0.469
BMI (+1)	1.02	0.95–1.10	0.539	0.99	0.92–1.09	0.950
AHI (≥30 vs. <30)	4.87	1.56–15.2	0.006	3.16	1.14–8.75	0.027
Gender (male vs. female)	0.43	0.13–1.43	0.168	0.87	0.22–3.35	0.842
Family and/or friends have positive experience with CPAP (yes vs. no)	4.29	1.05–17.51	0.042	1.11	0.45–2.73	0.827
Living with Partner (yes/no)	0.33	0.08–1.29	0.110	8.82	1.03–75.8	0.047
Tobacco Smoking (+1 packs years)	1.01	0.99–1.03	0.461	1.01	0.99–1.02	0.631
High Income (vs. Average Income)				0.36	0.12–1.05	0.062

AHI – apnea-hypopnea index, BMI – body mass index, CPAP – continuous positive airway pressure, CVD – cardiovascular disease, ESS – Epworth Sleepiness Scale, Financial Incentive – received financial support, HTN – hypertension.

Area under the ROC 81.0% and 69.3% for low income and average/high income patients, respectively.

### CPAP adherence

At one-year follow-up CPAP adherence (CPAP usage >4 hours/night, ≥6 day/week) was similar in the financial incentive and control groups, 35% and 39%, respectively (*p* = 0.82). Odds for CPAP adherence at one-year follow up among the financial incentive group were similar to the control group (0.86, 0.36–2.07). Multivariable analysis (adjusting for financial incentive, income level, age, gender, and tobacco smoking) revealed that the CPAP adherence at one year was sensitive to years of education (+1yr) (1.28, 1.06–1.55) and AHI (30 vs. <30) (5.25, 1.34–18.5), area under the ROC curve of 76.1%.

## Discussion

Financial incentive was associated with greater likelihood of CPAP acceptance among low-SES OSA patients in a health care system using a cost sharing strategy to contain costs. Therefore, we suggest that financial incentive should be applied as a policy to encourage CPAP acceptance, especially among low SES patients.

### Study strength and limitation

According to the Israeli National Health Insurance Law, all patients have free of charge access to PSG and CPAP titration studies [16], [26], [41], [42]. Both the control and financial incentive groups were recruited using a longitudinal study design. One possible shortcoming of our study was that we were not allowed by our Institutional Review Board to perform randomization of patients to groups and conduct a parallel study design, because patients were recruited during routine PSG study. We believe that this possibility of shortcoming is low, since both control and incentive groups were identical in all parameters (Table 1) except for tobacco smoking and T_90%_. Accordingly, our model was adjusted for tobacco smoking, which may be taken as a surrogate for poor health behavior. Moreover, similar CPAP pressure, AHI on CPAP, and side effects scores were found in the control and incentive groups. An additional possible limitation could be the self-reported income of the patient, particularly if individual income does not reflect overall household income, which may be more representative of SES. Finally, models were adjusted for time-dependent factors (time block) by recruiting additional 28 (30%) subjects who were found to be similar in all parameters to the 93 control subjects recruited at study initiation. In both groups, upon completion of the CPAP support patients declining CPAP experienced a greater side effects score. Multivariable logistic regression analysis revealed that financial incentive was an independent predictor of CPAP acceptance among low-SES patients.

In both groups, CPAP purchase was about 40% lower in young adult (≤40 years) OSA patients relative to older patients. In young adults with OSA, the prevalence of hypertension and cardiovascular disease is lower [41], [43]. It is possible that young adults with OSA will be less determined to commence CPAP when no symptoms exist, and they may seek other therapeutic options. In addition, clinicians′ attention and awareness preclude recommending CPAP treatment at younger ages [41].

### Cost incentive and CPAP treatment

To our knowledge this is the first study exploring the effect of financial incentive on patients′ acceptance of CPAP technology. In our study CPAP acceptance rates were unacceptably low, and only one-third of patients in the control group accepted the CPAP, supporting our previous findings [15], [16]. This result is in contrast to other reports demonstrating that when receiving support protocol, CPAP acceptance is about 70% [20], [21]; possible explanations for this gap were discussed elsewhere [16]. Minimization of cost sharing to $55 increases CPAP acceptance by 43.2%. Only 4.8% vs. 50% (*p* = 0.013) from the incentive and control groups, respectively, refused treatment claiming that cost was a substantial factor determining their decision. From an economic point of view, cost sharing is an administrative tool aimed to reduce health care costs and to prevent moral hazard. This policy may unfairly discourage people from seeking essential medical services and medication [28]–[30]. In practice, cost sharing is rarely linked to the service′s value [26], [28]–[32], [44] including CPAP [15], [16] especially among lower SES populations. In a chart review study, 42% of patients from minority-serving institutions diagnosed with OSA failed to follow up for treatment despite having medical insurance coverage for CPAP, compared with 7% in a voluntary hospital group [45]. Similar findings were reported among other urban patient populations [39], [46]. In low SES patients, CPAP purchase is low, despite having medical insurance for OSA diagnosis and CPAP titration study; it is possible that these patients are less knowledgeable about their disease and its treatment options [39], [45], [46]. Financial incentive was found to be an effective tool in minimizing health risk-behavior, particularly among low-SES people [33], [34], [47]. Implementing a large value-based insurance design program as offered by Blue Cross Blue Shield of North Carolina [48] and by a large employer′s value-based insurance [49], increases adherence rate to chronic essential medication. Not surprisingly, disease management per se was not sufficient to overcome the barrier of co-payment [49]. In our study adherence to CPAP one year after accepting a device was similar in both groups. Our findings support the notion that no single factor has been consistently identified as predictive of CPAP acceptance and adherence [3], [15], [16], [22], [24]. Understanding obstacles and critical elements associated with a patient′s decision to accept CPAP is crucial to promote successful treatment adherence. This includes optimal disease management, which improves patients′ attitudes, beliefs, and expectations about OSA and its treatment [17], [22]–[24], [50] in addition to minimizing cost sharing for CPAP.

In our study, AHI was an independent predictor that increased the odds for CPAP acceptance. Other studies have demonstrated that AHI has a weak relationship with CPAP treatment acceptance [3], mainly in symptomatic patients. It is possible that primary care physician recommendations to commence CPAP are based on AHI. However, Brin *et al*. [15] found a CPAP purchase rate of 35% after analyzing the subgroup of the most severe and symptomatic patients. It is possible that policy criteria only including AHI may affect physician recommendations to commence CPAP. The current study confirms Simon-Tuval′s [16] findings indicating that the primary care physician impact on patient decision to accept CPAP was probably minimal, i.e., our patients reported that little information was obtained from their physicians regarding therapeutic options. It is possible that the average/high-income patients did not respond to this change in cost-sharing policy since the amount of money saved in our cost sharing policy was negligible relative to their income level, and they may interpret this financial incentive as a means to encourage them to use an unneeded therapy [34].

Cost sharing is not a one-size-fits-all tool; it may be appropriate when health services are of low value and inappropriate when health services are of high value [34], as in the case of CPAP treatment. In Israel, co-payment is high and widely used across essential and non-essential treatments [30]. Our study suggests that adapting a more benefit-based cost sharing34 will provide a financial incentive for individuals to prioritize their out-of-pocket expenditures based on the value of their treatments, not their price. As a result, more balanced expenditure-control strategy will increase demand and will minimize discrimination of a vital health care service, mainly among vulnerable populations [34], [51], [52].

### Conclusions

Minimizing cost sharing reduces a major barrier for CPAP acceptance among low SES patients with OSA. Implementation of value-based cost sharing strategy for acceptance of a CPAP device would provide a positive financial incentive for patients with OSA to prioritize the option of CPAP treatments as a high-value care based on its potential clinical benefit.

## Supporting Information

Methods S1 **For additional description of the methods see Methods S1 section.**
(DOC)

## References

[1] Punjabi NM (2008). The epidemiology of adult obstructive sleep apnea.. Proc Am Thor Soc.

[2] Kushida CA, Morgenthaler TI, Littner MR, Alessi CA, Bailey D (2006). American Academy of Sleep Medicine. Practice parameters for the use of continuous and bilevel positive airway pressure devices to treat adult patients with sleep-related breathing disorders.. Sleep.

[3] Gay P, Weaver T, Loube D, Iber C (2006). Positive Airway Pressure Task Force; Standards of Practice Committee; American Academy of Sleep Medicine. Evaluation of positive airway pressure treatment for sleep related breathing disorders in adults.. Sleep.

[4] Weaver TE, Maislin G, Dinges DF, Bloxham T, George CF (2007). Relationship between hours of CPAP use and achieving normal levels of sleepiness and daily functioning.. Sleep.

[5] McArdle N, Kingshott R, Engleman HM, Mackay TW, Douglas NJ (2001). Partners of patients with sleep apnoea/hypopnoea syndrome: effect of CPAP treatment on sleep quality and quality of life.. Thorax.

[6] Marin JM, Carrizo SJ, Vicente E, Agusti AG (2005). Long-term cardiovascular outcomes in men with obstructive sleep apnoea-hypopnoea with or without treatment with continuous positive airway pressure: an observational study.. Lance.

[7] McDaid C, Duree KH, Griffin SC, Weatherly HL, Stradling JR (2009). An economic analysis of continuous positive airway pressure for the treatment of obstructive sleep apnea-hypopnea syndrome.. Int J Technol Assess Health Care.

[8] McDaid C, Griffin S, Weatherly H, Durée K, van der Burgt M (2009). Continuous positive airway pressure devices for the treatment of obstructive sleep apnoea-hypopnoea syndrome: a systematic review and economic analysis.. Health Technol Assess 13:iii-iv, xi-v, 1–119,.

[9] Guest JF, Helter MT, Morga A, Stradling JR (2008). Cost-effectiveness of using continuous positive airway pressure in the treatment of severe obstructive sleep apnoea/hypopnoea syndrome in the UK.. Thorax.

[10] Ayas NT, FitzGerald JM, Fleetham JA, White DP, Schulzer M (2006). Cost-effectiveness of continuous positive airway pressure therapy for moderate to severe obstructive sleep apnea/hypopnea.. Arch Intern Med.

[11] Ip MS, Tse HF, Lam B, Tsang KW, Lam WK (2004). Endothelial function in obstructive sleep apnea and response to treatment.. Am J Respir Crit Care Med.

[12] Martínez-García MA, Soler-Cataluña JJ, Ejarque-Martínez L, Soriano Y, Román-Sánchez P (2009). Continuous positive airway pressure treatment reduces mortality in patients with ischemic stroke and obstructive sleep apnea: a 5-year follow-up study.. Am J Respir Crit Care Med.

[13] Marti S, Sampol G, Muñoz X, Torres F, Roca A (2002). Mortality in severe sleep apnoea/hypopnoea syndrome patients: impact of treatment.. Eur Respir J.

[14] Albarrak M, Banno K, Sabbagh AA, Delaive K, Walld R (2005). Utilization of healthcare resources in obstructive sleep apnea syndrome: a 5-year follow-up study in men using CPAP.. Sleep.

[15] Brin YS, Reuveni H, Greenberg-Dotan S, Tal, A, Tarasiuk A (2005). Determinants affecting initiation of continuous positive airway pressure treatment.. Isr Med Assoc J.

[16] Simon-Tuval T, Reuveni H, Greenberg-Dotan S, Oksenberg A, Tal A (2009). Low socioeconomic status is a risk factor for CPAP acceptance among adult OSAS patients requiring treatment.. Sleep.

[17] Olsen S, Smith S, Oei T, Douglas J (2008). Health belief model predicts adherence to CPAP before experience with CPAP.. Eur Respir J.

[18] Reuveni H, Tarasiuk A, Wainstock T, Ziv A, Elchayani A (2004). Awareness level to obstructive sleep apnea syndrome during routine unstructured interviews of a standardized patient by primary care physicians.. Sleep.

[19] Lettieri CJ, Shah AA, Holley AB, Kelly WF, Chang AS (2009). Effects of a short course of eszopiclone on continuous positive airway pressure adherence: a randomized trial.. Ann Intern Med.

[20] Hoy CJ, Vennelle M, Kingshott RN, Engleman HM, Douglas NJ (1999). Can intensive support improve continuous positive airway pressure use in patients with the sleep apnea/hypopnea syndrome?. Am J Respir Crit Care Med.

[21] Popescu G, Latham M, Allgar V, Elliott MW (2001). Continuous positive airway pressure for sleep apnea/hypopnoea syndrome: usefulness of a 2-week trial to identify factors associated with long term use.. Thorax.

[22] Weaver TE, Grunstein RR (2008). Adherence to continuous positive airway pressure therapy: the challenge to effective treatment.. Proc Am Thorac Soc.

[23] Stepnowsky CJ, Bardwell WA, Moore PJ, Ancoli-Israel S, Dimsdale JE (2002). Psychologic correlates of compliance with continuous positive airway pressure.. Sleep.

[24] Aloia MS, Arnedt JT, Stepnowsky C, Hecht J, Borrelli B (2005). Predicting treatment adherence in obstructive sleep apnea using principles of behavior change.. J Clin Sleep Med.

[25] Mackenbach JP, Stirbu I, Roskam AJ, Schaap MM, Menvielle G (2008). European Union Working Group on Socioeconomic Inequalities in Health. Socioeconomic inequalities in health in 22 European countries.. N Engl J Med.

[26] Tarasiuk A, Greenberg-Dotan S, Simon T, Tal A, Oksenberg A (2006). Low socioeconomic status is a risk factor for cardiovascular disease among adult OSAS patients requiring treatment.. Chest.

[27] Alter DA, Naylor CD, Austin P, Tu JV (1999). Effects of socioeconomic status on access to invasive cardiac procedures and on mortality after acute myocardial infarction.. N Engl J Med.

[28] Goldman DP, Joyce GF, Zheng Y (2007). Prescription drug cost sharing: associations with medication and medical utilization and spending and health.. JAMA.

[29] Heisler M, Langa KM, Eby EL, Fendrick AM, Kabeto MU (2004). The health effects of restricting prescription medication use because of cost.. Med Care.

[30] Reuveni H, Sheizaf B, Elhayany A, Sherf M, Limoni Y (2002). The effect of drug co-payment policy on the purchase of prescription drugs for children with infections in the community.. Health Policy.

[31] Goldman DP, Joyce GF, Escarce JJ, Pace JE, Solomon MD (2004). Pharmacy benefits and the use of drugs by the chronically ill.. JAMA.

[32] Choudhry NK, Avorn J, Antman EM, Schneeweiss S, Shrank WH (2007). Should patients receive secondary prevention medications for free after a myocardial infarction? An economic analysis.. Health Aff (Millwood).

[33] Giuffrida A, Torgerson DJ (1997). Should we pay the patient? Review of financial incentives to enhance patient compliance.. BMJ.

[34] Fendrick AM, Smith DG, Chernew ME, Shah SN (2001). A benefit-based copay for prescription drugs: patient contribution based on total benefits, not drug acquisition cost.. Am J Manag Care.

[35] Fendrick AM (2010). Value-based insurance design for diabetes mellitus: approaches to optimal pharmacoeconomic implementation.. Am J Manag Care.

[36] Mahoney JJ (2005). Reducing patient drug acquisition costs can lower diabetes health claims.. Am J Manag Care.

[37] Johns MW (1991). A new method for measuring daytime sleepiness: the Epworth sleepiness scale.. Sleep.

[38] Kump K, Whalen C, Tishler PV, Browner I, Ferrette V (1994). Assessment of the validity and utility of a sleep-symptom questionnaire.. Am J Respir Crit Care Med.

[39] Kribbs NB, Pack AI, Kline LR, Smith PL, Schwartz AR (1993). Objective measurement of patterns of nasal CPAP use by patients with obstructive sleep apnea.. Am Rev Respir Dis.

[40] Smith S, Lang C, Sullivan K, Warren J (2004). Two new tools for assessing patients′ knowledge and beliefs about obstructive sleep apnea and continuous positive airway pressure therapy.. Sleep Med.

[41] Reuveni H, Greenberg-Dotan S, Simon-Tuval T, Oksenberg A, Tarasiuk A (2008). Young Adult Males with OSA Consume High Health Care Resources due to Nonspecific Co-Morbidity.. Eur Respir J.

[42] Tarasiuk A, Greenberg-Dotan S, Simon T, Tal A, Oksenberg A (2008). Elderly with Obstructive Sleep Apnea Consume High Health Care Resources due to elevated cardiovascular morbidity.. J Am Geriatr Soc.

[43] Nieto FJ, Young TB, Lind BK, Shahar E, Samet JM (2000). Association of sleep-disordered breathing, sleep apnea, and hypertension in a large community-based study. Sleep Heart Health Study.. JAMA.

[44] DiMatteo MR (2004). Variations in patients′ adherence to medical recommendations: a quantitative review of 50 years of research.. Med Care.

[45] Greenberg H, Fleischman J, Gouda HE, De La Cruz AE, Lopez R (2004). Disparities in obstructive sleep apnea and its management between a minority-serving institution and a voluntary hospital.. Sleep Breath.

[46] Platt AB, Field SH, Asch DA, Chen Z, Patel NP (2009). Neighborhood of residence is associated with daily adherence to CPAP therapy.. Sleep.

[47] Sutherland K, Christianson JB, Leatherman S (2008). Impact of targeted financial incentives on personal health behavior: a review of literature.. Med Care Res Rev Suppl.

[48] Maciejewski ML, Farley JF, Parker J, Wansink D (2010). Copayment reductions generate greater medication adherence in targeted patients.. Health Aff (Millwood).

[49] Chernew ME, Shah MR, Wegh A, Rosenberg SN, Juster IA (2008). Impact of decreasing copayments on medication adherence within a disease management environment.. *Health Aff* (Millwood).

[50] Bandura A (1986). Social Foundations of thought and action: a social cognitive theory..

[51] Chernew ME, Juster IA, Shah M, Wegh A, Rosenberg S (2010). Evidence that value-based insurance can be effective.. Health Aff (Millwood).

[52] Braithwaite RS, Omokaro C, Justice AC, Nucifora K, Roberts MS (2010). Can broader diffusion of value-based insurance design increase benefits from US health care without increasing costs? Evidence from a computer simulation model.. PLoS Med.

