# Supplementary material for: Financial Incentive Increases CPAP Acceptance in Patients from Low Socioeconomic Background
Source: PLoS One. 2012 Mar 30;7(3):e33178. doi: 10.1371/journal.pone.0033178 (PMC3316560; doi:10.1371/journal.pone.0033178)
Supplement: Methods S1 — For additional description of the methods see Methods S1 section. (DOC) [file pone.0033178.s001.doc]

##### Methods S1

Financial Incentive Increases CPAP Acceptance in Patients

**from Low Socioeconomic Background**

Ariel Tarasiuk, Gally Reznor, Sari Greenberg-Dotan, Haim Reuveni

**METHODS**

**Cost sharing**: Patients in the control group were required to pay the full cost for the CPAP treatment. The financial incentive group was offered CPAP at a subsidized price. In cases where auto-titrating CPAP was purchased, patients in both groups were required to pay the difference up to $500.

Participants in both groups were recruited prior to the polysomnographic (PSG) study. Following a brief explanation by study interviewers, they were provided with a prepared script informing them about the study’s aims and what the study protocol requires from them. They were informed that: “…this study explores determinants affecting a patient’s decision to accept continuous positive airway pressure (CPAP) …. the treatment of choice for obstructive sleep apnea…”; and “…following receipt of their results with a recommendation for CPAP treatment, we will enlist them if they consent”. Both (control and financial incentive) groups were informed that CPAP treatment requires mandatory out-of-pocket payment according to the Israel National Health Insurance Law; the incentive group was told that they will pay only a subsidized price of $55 for their CPAP device (see Cost sharing section of the Methods in the main text). The Human Subject Committee of Soroka University Medical Center approved protocol number 10262.

For CPAP support we used a modification of the approach previously described by Popescu et al [S1]. All patients participated in a pre-adaptation session in the sleep laboratory with a sleep technician, during which patients received further explanation of the pathophysiology of OSAS, its risks and treatment, and an explanation of the upcoming adaptation trial. A two-week period of adaptation with an autotitrating CPAP device at home was offered and encouraged. During the pre-adaptation period, patients were fitted with an appropriate mask selected from a variety of CPAP devices and masks, and were given a humidifier if needed. Following that, the patient was guided as to how to use the device at home and received a brochure highlighting helpful tips regarding common problems with CPAP use. During the adaptation trial, when patients used the device at home, a technologist was in contact with the patient by phone or laboratory appointment at least once a week as needed to solve problems. Patients were encouraged to try a variety of masks and CPAP manufacturers free of charge. Four to six weeks following the conclusion of the adaptation period, the final decision of our patients to commence (purchase) or decline CPAP treatment was confirmed.

**Polysomnographic study** (PSG): Overnight PSG was performed according to previously described methods [S2]. Subjects reported to the laboratory at 8:30 PM and were discharged the following morning. Subjects were encouraged to maintain their usual daily routine and to avoid any caffeine and/or alcohol intake on the day of the study. Shift workers did not perform the PSG study in the week following shift duty. PSG study included electroencephalography (EEG), electrooculography (EOG), electromyography (EMG) applied over the submental muscles and bilateral anterior tibialis muscles for detection of periodic limb movements, electrocardiography (ECG), respiratory activity (airflow and chest/abdominal effort), body position, and oxygen saturation (SaO_2_). The percent-sleeping time in which oxygen saturation was below 90% (T_90_) was determined. Scoring was done by a trained technician using the traditional criteria [S3], reviewed by a polysomnographer, and a report sent to the referring physician. Obstructive apnea was defined as an episode of complete cessation of breathing (airflow reduction of >80%) of ≥10 seconds with continuing inspiratory effort. A hypopnea was scored when continuing inspiratory effort was accompanied by a reduction of at least 50% in airflow, resulting in either an arousal or oxygen desaturation of at least 4%. The AHI was calculated as the number of respiratory events (apnea/hypopnea) per hour of sleep. A sleep physician explained the significance of the results to the patients (prior to the CPAP support program) with recommendations for treatment. CPAP titration was performed during an approximately two-week period following PSG study using attendant autotitration CPAP protocol.

Prior to the PSG study, we asked [S4]: “Does your partner sleep in a separate room, on regular basis, due to your snoring problems? Yes/no”; “Did your primary care physician explain to you what medical problem/s could be diagnosed in a sleep study? Yes/no/not enough”; “Did your primary care physician explain to you what obstructive sleep apnea syndrome is and what treatments options are available? Yes/no”; “Did you receive information from a person being treated with CPAP? Yes/no”; “Was his/her experience with CPAP positive/negative/neutral?” At the conclusion of the CPAP support we explored reasons for CPAP acceptance, e.g., “CPAP solved my snoring problems, yes/no”; “CPAP treatment reduced my excessive daytime sleepiness, yes/no”; “CPAP treatment improved my sleep, yes/no”; “Do you think CPAP treatment will improve your risks of further morbidities (e.g., CVD, HTN)? yes/no/don’t know”; “Do you acknowledge the fact that CPAP treatment is the best treatment available for you? yes/no”. Reasons for declining CPAP treatment included whether the following options were applicable to them: “I am interested in other treatment options”; “I could not adapt to CPAP”; “I feel better and don’t need this treatment”; “I was not encouraged to commence CPAP by my partner/my physician/family/friend”; “I cannot purchase CPAP since it is too expensive for me (yes/no)”. We also collected information regarding post-treatment AHI and mask leak.

Socioeconomic status was evaluated according to self-reported monthly income and Israel Central Bureau of Statistics data [S4,S5]. We reviewed the accumulated diagnoses along with their respective International Classification of Diseases, Ninth Revision (ICD-9) codes. [S5] including hypertension [401–405], ischemic heart disease [410–414], cardiac arrhythmia, congestive heart failure, valvular cardiac disease, cerebrovascular accident [426–438], and peripheral vascular disease [443]. CVD diagnosis [S6] included at least one of the following ICD-9 codes: 410–414, 426–438, and 443.

**Data and statistical analysis**: All data were analyzed using SPSS Software (v 17). Continuous variables are presented as mean with standard deviation, unless specified otherwise. To compare differences in study variables we used independent-sample *t*-tests for normal distribution variables, Mann-Whitney U and Wilcoxon signed-rank tests for non-parametric independent variables, and Chi-Square tests for nominal variables. Logistic regression analysis was used to investigate factors influencing CPAP acceptance 4 to 6 weeks following conclusion of the adaptation period. Independent variables included age, gender, HTN, CVD, smoking, monthly income level, PSG findings (AHI, T_90%_), ESS, and support from family and/or friends. Models were also adjusted for time-dependent factors (time-block) by recruiting an additional 28 (30%) subjects at the conclusion of the study. The area under the receiver operating characteristic (ROC) curve was calculated for the model. The null hypothesis was rejected at the 5% level.

##### On-Line References

S1. Popescu G, Latham M, Allgar V, Elliott MW (2001) Continuous positive airway pressure for sleep apnea/hypopnoea syndrome: usefulness of a 2-week trial to identify factors associated with long term use. Thorax;56:727-33.

S2. Rotem AY, Sperber AD, Krugliak P, Freidman B, Tal A, et al. (2003) Polysomnography and actigraphy evidence of sleep fragmentation in irritable bowel syndrome. Sleep;26:746-52.

S3. Sleep-related breathing disorders in adults: recommendations for syndrome definition and measurement techniques in clinical re­search. (1999) The Report of an American Academy of Sleep Medicine Task Force. Sleep;22:667-89.

S4. Simon-Tuval T, Reuveni H, Greenberg-Dotan S, Oksenberg A, Tal A, et al. (2009) Low socioeconomic status is a risk factor for CPAP acceptance among adult OSAS patients requiring treatment. Sleep;32:545-52.

S5. Statistical abstract of Israel. No 53. Tables 12 and 37. Central Bureau of Statistics.

State of Israel, 2002.

S6. Tarasiuk A, Greenberg-Dotan S, Simon T, Tal A, Oksenberg A, et al. (2006) Low socioeconomic status is a risk factor for cardiovascular disease among adult OSAS patients requiring treatment. Chest;130:766-73.
